# Supplementary material for: The Quenching of Long-Wavelength Fluorescence by the Closed Reaction Center in Photosystem I in Thermostichus vulcanus at 77 K
Source: Int J Mol Sci. 2024 Nov 19;25(22):12430. doi: 10.3390/ijms252212430 (PMC11594324; doi:10.3390/ijms252212430)
Supplement: Supplementary file 1 [file ijms-25-12430-s001.zip › ijms-3292251-supplementary.pdf]

Quenching of the long-wavelength fluorescence by the closed reaction centre of Photosystem I of *Thermostichus vulcanus* at 77 K

Parveen Akhtar, Ivo H.M. van Stokkum, and Petar H. Lambrev

Supplementary Material

Method S1. Steady-state fluorescence spectroscopy

Fluorescence emission spectra in the visible range were measured at RT and 77 K using a FP-8500 (Jasco, Japan) spectrofluorometer. The sample was diluted to an absorbance of 0.1 per cm at the red maximum cooled in a home-built accessory used with the FP-8500 spectrofluorometer. Emission spectra in the range of 620–780 nm were recorded with excitation wavelength of 440 nm and excitation/emission bandwidth of 2.5 nm. The measurements were performed with 1 nm increment and 4 s integration time. The spectra were corrected for the spectral sensitivity of the instrument using a calibrated light source (ESC-842, Jasco) as a reference.

The fluorescence emission spectra recorded at RT and 77 K are presented in Fig. S1. The RT emission spectrum reveals two main peaks - one at 680 nm originating from the bulk antenna Chls, and another at 725 nm from low-energy, Red Chls. These Red Chls are crucial in capturing and transferring light to the Rc, thereby enhancing the efficiency of PSI in the light-harvesting process. At 77 K, the spectral features shift significantly, showing a pronounced peak at 731 nm, indicating enhanced emission from the Red Chls. The reduction in thermal energy at low temperatures slows redistribution of excitations between energy states, which in turn allows the Red Chls to emit more efficiently. The low-temperature condition reveals the critical contribution of these low-energy states to the overall fluorescence of PSI.

In addition to the prominent 731 nm peak at 77 K, lower-intensity emission bands below 700 nm—representing the bulk antenna Chls—are still visible, but less intense compared to the RT spectrum. In addition to the dominant 731 nm peak at 77 K, low-intensity emission bands below 700 nm are also visible, representing the bulk antenna Chls. These peaks are less pronounced compared to the RT

spectrum, reflecting the decreased efficiency of EET from the bulk Chls to the Red Chls at low temperatures.

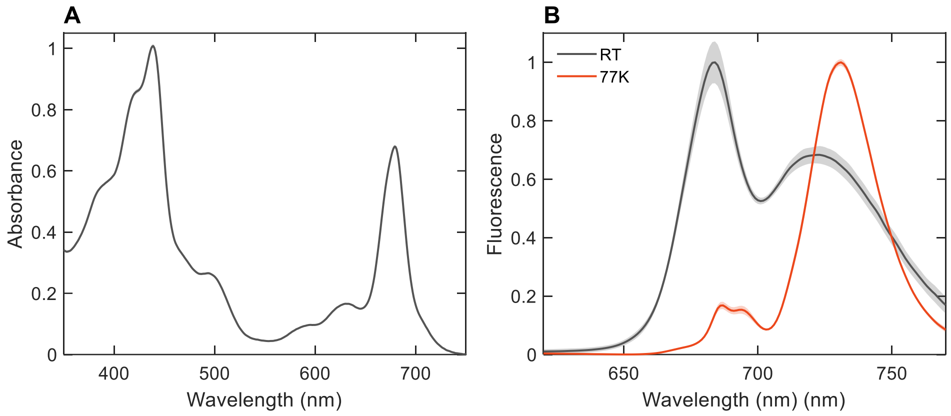

Figure S 1. Absorption and fluorescence emission spectra of PSI trimers of T. vulcanus measured in buffer containing 0.03% β-DM. (A) Room temperature absorption spectra and (B) Fluorescence emission spectra at room temperature and 77 K with 440 nm excitation light, normalized to the maximum. The figures show average spectra from 4 independent measurements on different batches with standard errors represented by the shaded area.

Experimental and model time gated spectra and kinetic traces

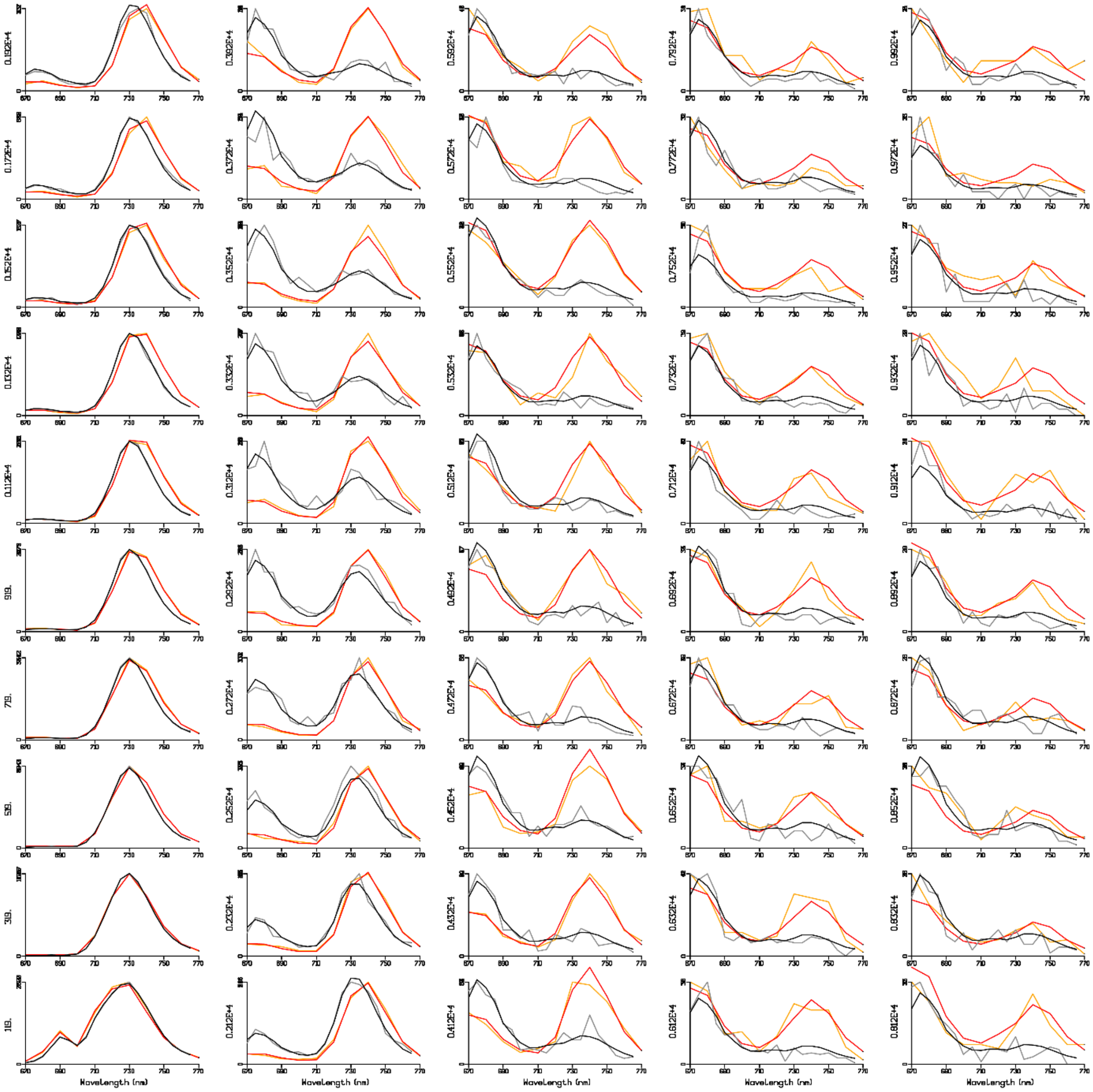

Figure S 2. Time gated spectra of the PSI emission at 50 decay times (in steps of 200 ps, starting from the bottom left to the top right, indicated in the ordinate labels of the panels) after 440 nm excitation at 77K. Grey (orange) and black (red) lines indicate the data and the global analysis fit, of the PSI in the closed and "open" RC experiments, respectively. Note also that each panel is scaled to its maximum.

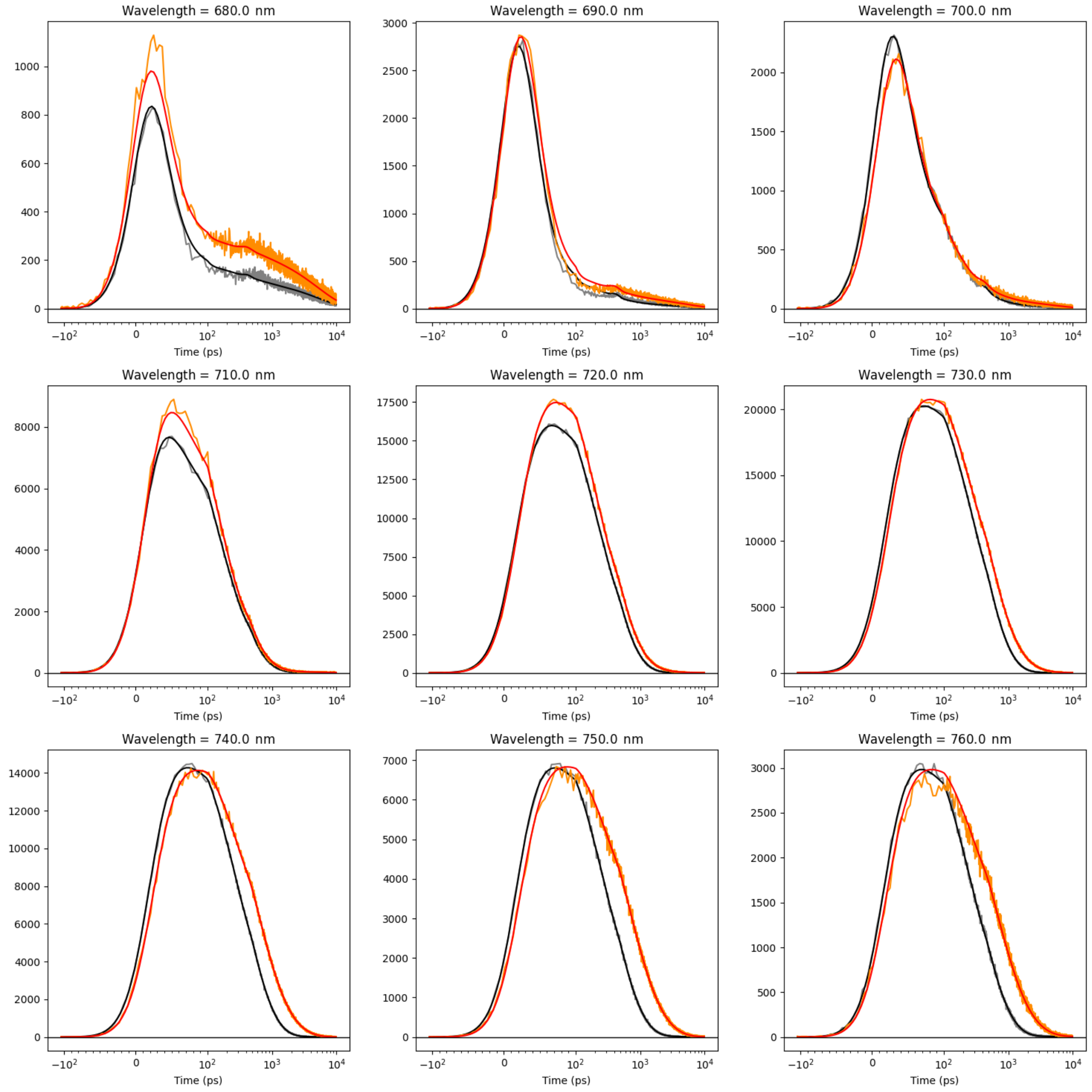

Figure S 3. Time traces of the PSI emission at 9 wavelengths (indicated in the title of the panels) after 440 nm excitation at 77 K. Grey (orange) and black (red) lines indicate the data and the target analysis fit, of the PSI in the closed and "open" RC experiments, respectively. Note that the time axis is linear until 100 ps and logarithmic thereafter. Note also that each panel is scaled to its maximum. The overall rms error of the fit was 18.3.

Amplitude matrices

Table S1 Complex ‘b’ closed RC

**species**<br>**initial concentration**<br>**lifetime↓**	**Bulk**<br>**1**	**Red 2b**<br>**0.020**	**Red 1**<br>**0.040**	**Sum**
13.6 ps	0.889	-0.304	-0.035	0.549
108 ps	0.009	-0.034	0.067	0.042
462 ps	0.012	0.356	0.004	0.372
Sum	0.909	0.018	0.036	0.964

Table S2 Complex ‘c’ closed RC

**species**<br>**initial concentration**<br>**lifetime↓**	**Bulk**<br>**1**	**Red 2c**<br>**0.020**	**Red 1**<br>**0.040**	**Sum**
14.8 ps	0.893	-0.272	-0.039	0.582
109 ps	0.011	-0.079	0.071	0.003
155 ps	0.005	0.369	0.004	0.379
Sum	0.909	0.018	0.036	0.964

Table S3 Complex ‘a’ open or closed RC

**species**<br>**initial concentration**<br>**lifetime↓**	**Bulk**<br>**1**	**Red 2a**<br>**0.020**	**Red 1**<br>**0.040**	**Sum**
14.7 ps	0.879	-0.249	-0.038	0.592
108 ps	0.008	-0.032	0.066	0.042
310 ps	0.022	0.299	0.009	0.330
Sum	0.909	0.018	0.036	0.964

Table S4 Complex ‘b’ open RC

species<br>initial concentration<br>lifetime↓	Bulk<br>1	Red 2b<br>0.020	Red 1<br>0.040	Sum
13.6 ps	0.889	-0.302	-0.035	0.552
108 ps	0.009	-0.032	0.068	0.045
615 ps	0.012	0.352	0.004	0.367
Sum	0.909	0.018	0.036	0.964

Table S5 Complex ‘c’ open RC

species<br>initial concentration<br>lifetime↓	Bulk<br>1	Red 2c<br>0.020	Red 1<br>0.040	Sum
14.8 ps	0.894	-0.249	-0.039	0.606
109 ps	0.012	-0.026	0.074	0.060
1380 ps	0.003	0.294	9.9e-04	0.298
Sum	0.909	0.018	0.036	0.964
